# Supplementary material for: MAPS: pathologist-level cell type annotation from tissue images through machine learning
Source: Nat Commun. 2024 Jan 2;15:28. doi: 10.1038/s41467-023-44188-w (PMC10761896; doi:10.1038/s41467-023-44188-w)
Supplement: Supplementary file 1 — Supplementary Information [file 41467_2023_44188_MOESM1_ESM.pdf]

Supplementary Information for

MAPS: Pathologist-level cell type annotation from tissue images through machine learning

Muhammad Shaban*, Yunhao Bai*, Huaying Qiu*, Shulin Mao, Jason Yeung, Yao Yu Yeo, Vignesh Shanmugam, Han Chen, Bokai Zhu, Jason L. Weirather, Garry P. Nolan, Margaret A. Shipp, Scott J. Rodig, Sizun Jiang†, Faisal Mahmood†

*These authors contributed equally to this work.

†These authors jointly supervised this work.

† Corresponding author(s). E-mail(s): sjiang3@bidmc.harvard.edu; faisalmahmood@bwh.harvard.edu

This PDF file includes:

Supplementary Figures 1 to 4

Supplementary Tables 1 to 15

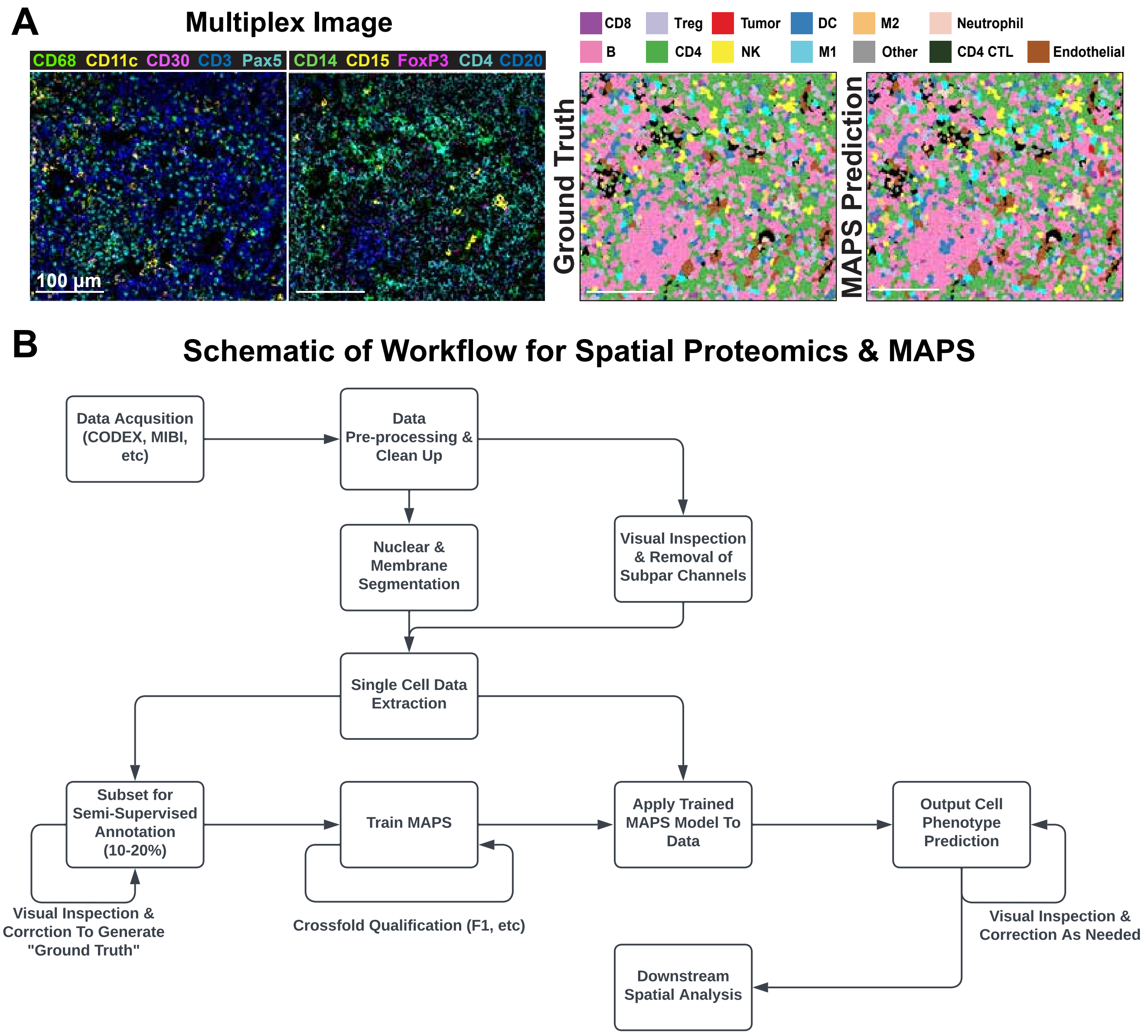

Supplementary Figure 1: Performance of MAPS on rLN and scheme of the MAPS workflow, related to Figure 1. (A) Representative FOV of a multiplexed image of rLN used for cell phenotyping. Cell phenotype maps generated via manual annotation (Ground Truth) or MAPS (MAPS Prediction) are shown for visual comparison. **(B)** Schematic of the workflow for spatial proteomics cell phenotyping accelerated by MAPS.

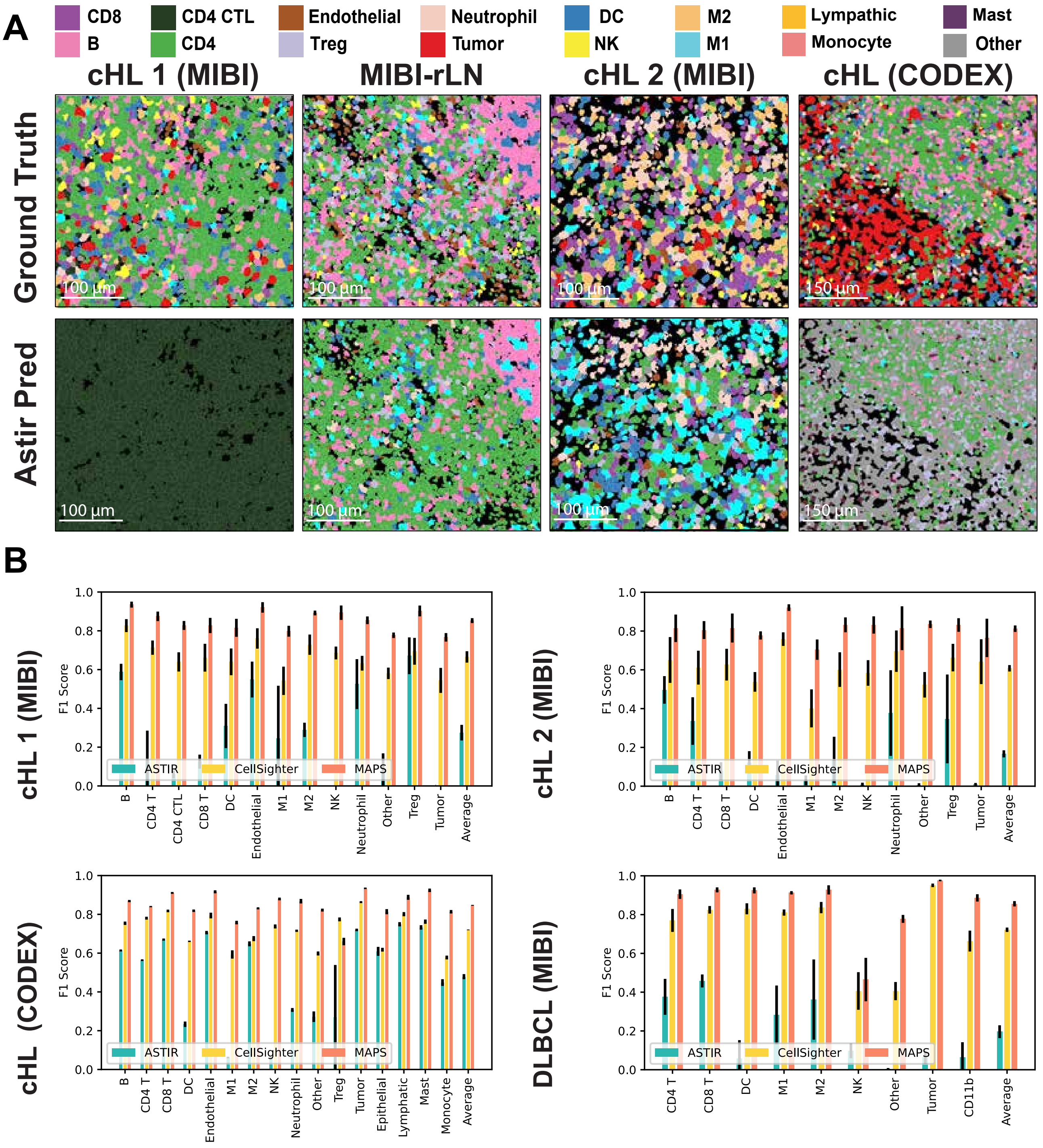

Supplementary Figure 2: Visual and quantitative comparison of MAPS performance with its counterparts, related to Figure 2. **(A)** Comparison of ground truth and Astir performances across four multiplex image datasets. **(B)** Performance comparison at class level F1-score of three cell phenotyping methods across all four datasets with average F1-score across 5-folds. Error bars represent ± 1 standard deviation. Source data are provided as a Source Data file.

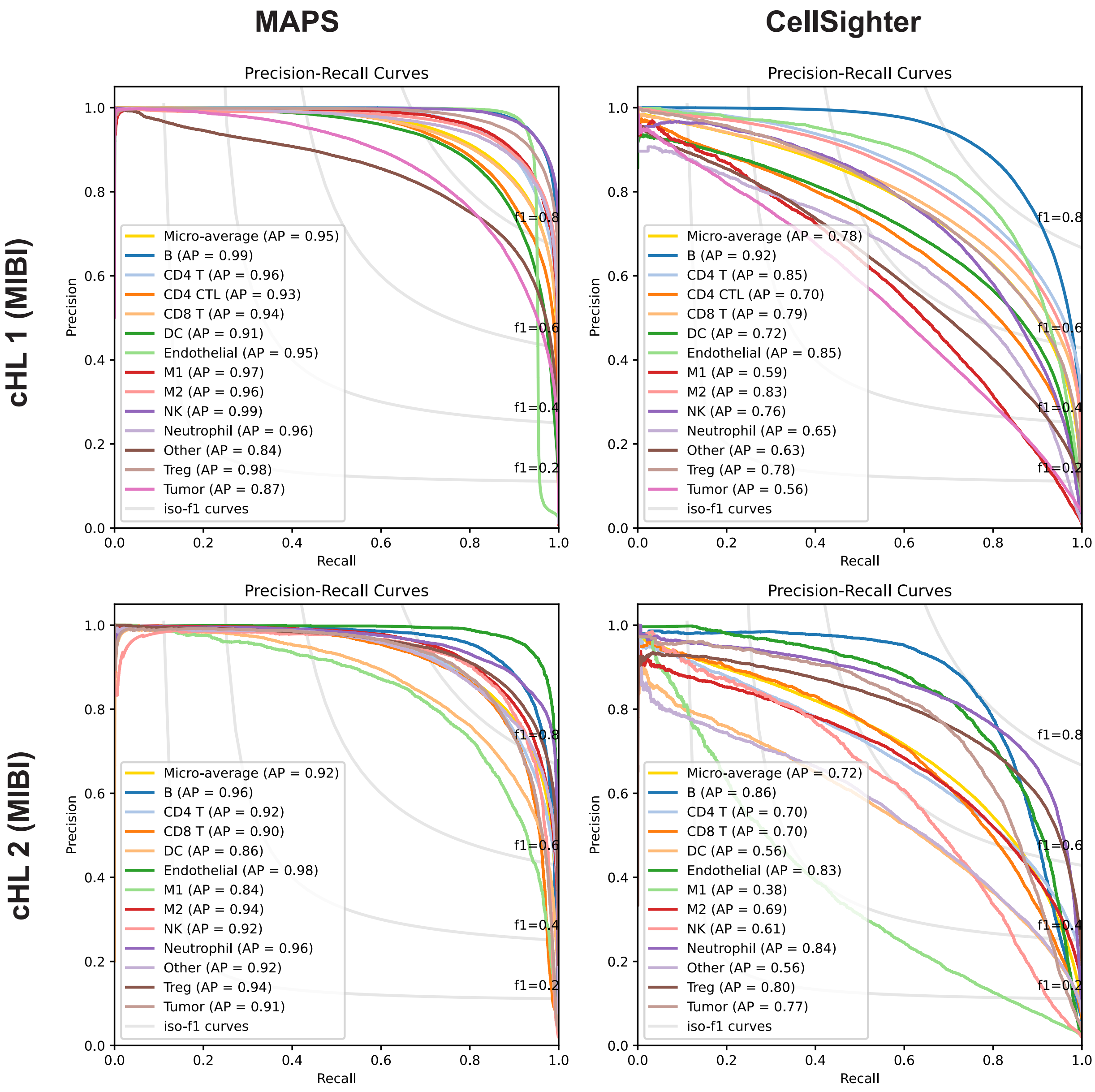

Supplementary Figure 3: Visual and quantitative comparison of MAPS performance with its counterparts. Precision and recall curves of MAPS and CellSighter based on the prediction for dataset cHL1 and cHL2. Each plot presents not only the overall precision and recall curve of the model but also the curves for each cell type. Each curve shows the precision and recall trade-off for different thresholds (Figure continued by the Supplementary Figure 4). Source data are provided as a Source Data file.

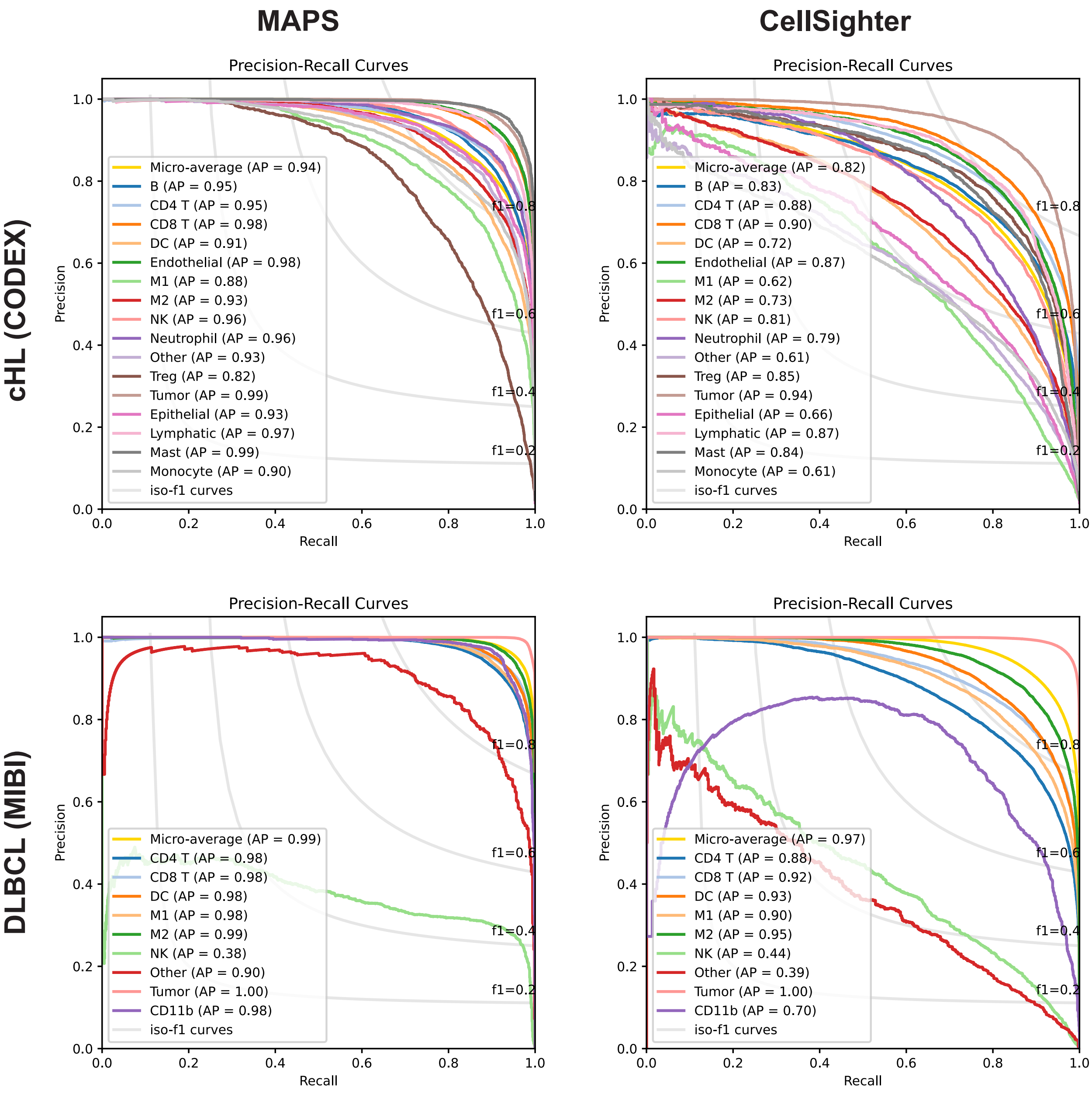

Supplementary Figure 4: Visual and quantitative comparison of MAPS performance with its counterparts. Precision and recall curves of MAPS and CellSighter based on the prediction for dataset cHL (CODEX) and DLBCL (MIBI). Each plot presents not only the overall precision and recall curve of the model but also the curves for each cell type. Each curve shows the precision and recall trade-off for different thresholds. Source data are provided as a Source Data file.

Supplementary Table 1. MIBI panel for cHL 1 and cHL 2 (MIBI)

Antibody target	Clone	Vendor	Catalog number	Mass	Isotope	Titer (µg/mL)	Note
CD45	2B11 PD7/26	Novus Bio	NBP2-34528	69	Ga	2.00	
CD20	rIGEL/773	Novus Bio	NBP2-53190	71	Ga	1.00	
dsDNA	35I9 DNA	Abcam	ab27156	89	Y	2.00	Not in cHL 1
pSLP-76	E3G9U	CST	76384	110	Cd	2.50	Not in cHL 1
SLP-76	D1R1A	CST	70896	111	Cd	3.00	Not in cHL 1
anti-H2AX (pS139)	N1-431	Abcam	ab303656	112	Cd	4.00	Not in cHL 1
CD163	EDHu-1	Novus Bio	NB110-40686	113	In	3.00	
Histone H3	D1H2	CST	4499	115	In	2.00	
CD45RO	UCHL1	BioLegend	304202	139	La	1.00	
CD28	D2Z4E	CST	38774S	140	Ce	2.50	
CD153 (CD30L)	Poly	R&D	AF1028	141	Pr	0.15	
Lag3	17B4	Novus Bio	NBP1-97657	142	Nd	0.50	
CD4	EPR6855	Abcam	ab181724	143	Nd	1.00	
CD11c	EP1347Y	Abcam	ab216655	144	Nd	0.50	
CD56	MRQ-42	Cell Marque	156R	145	Nd	0.60	
FoxP3	236A/E7	Abcam	ab96048	146	Nd	5.00	
GATA3	L50-823	Cell Marque	390M	147	Sm	5.00	
Granzyme B	EPR20129-127	Abcam	ab219803	148	Nd	1.00	
PD-L1	E1L3N	CST	13684	149	Sm	5.00	
CD16	SP175	Abcam	ab243925	150	Sm	1.00	
Ki-67	8D5	Novus Bio	NBP2-22112	151	Eu	0.25	
PD-1	D4W2J	CST	86163S	152	Sm	2.00	
PD-1	NAT105	Abcam	ab201811	152	Sm	2.00	
Pax-5	D7H5X	CST	93009	153	Eu	1.00	
Tox	E6I3Q	CST	73758	154	Sm	1.00	
CD161	Poly	Abcam	ab197979	155	Gd	3.00	
CD68	D4B9C	CST	26042	156	Gd	0.50	
B2-Microglobulin	D8P1H	CST	12851	157	Gd	4.50	
CD8	C8/144B	Cell Marque	108M	158	Gd	0.40	
CD3	MRQ-39	Cell Marque	103R	159	Gd	0.50	
HLA1	EMR8-5	Abcam	ab70328	160	Gd	0.33	
CD15	MC480	BioLegend	125602	161	Dy	1.50	
Tbet	4B10	BioLegend	644802	162	Dy	2.00	
CD14	SP192	Abcam	ab230903	163	Dy	0.50	
CD123	7G3	BD	554527	164	Dy	1.00	Not in cHL 1
CXCR5	EPR8837	Abcam	ab271898	165	Ho	1.00	
CD45RA	HI100	BioLegend	304102	166	Er	0.80	
HLA-DR	EPR3692	Abcam	ab92511	167	Er	0.60	
CD57	HNK-1	BioLegend	359602	168	Er	0.60	
IL-10	4A7-25-17	Abcam	ab134742	169	Tm	0.50	
CD30	Ber-H2	Cell Marque	130M	170	Er	1.00	
TIM3	D5D5R	CST	45280	171	Yb	1.00	
RORgT	AFKJS-9	Invitrogen	14-6988-82	172	Yb	1.00	
TCRgd	H-41	SCBT	sc-100289	173	Yb	1.00	
CD86	E2G8P	CST	91882	174	Yb	1.50	
CD25	4C9	Cell Marque	125M	175	Lu	1.00	
Na-K ATPase	EP1845Y	Abcam	ab167390	176	Yb	5.00	

Supplementary Table 2. CODEX panel for cHL

Antibody target	Clone	Vendor	Catalog number	Oligo	Cycle	Fluor	Titer (dilution)
BCL-2	124	Novus Bio	NBP2-34443	41	18	Cy3	1:50
CCR6	polyclonal	Novus Bio	NBP1-88565	53	4	Cy5	1:25
CD11b	EPR1344	Abcam	ab209970	28	19	Cy5	1:50
CD11c	EP1347Y	Abcam	ab216655	49	11	Cy3	1:50
CD15	MMA	BD	559045	14	23	Cy3	1:200
CD16	D1N9L	CST	72204	26	12	Cy3	1:100
CD162	HECA-452	Novus Bio	NB100-78039	46	25	Cy5	1:200
CD163	EDHu-1	Novus Bio	NB110-40686	45	21	Cy3	1:200
CD2	RPA-2.10	Biolegend	300202	25	5	Cy5	1:25
CD20	rIGEL/773	Novus Bio	NBP2-53190	48	10	Cy3	1:200
CD206	polyclonal	R&D Systems	AF2535	55	14	Cy3	1:100
CD25	4C9	Cell Marque	125M	24	9	Cy3	1:100
CD30	BerH2	Cell Marque	130M	57	5	Cy3	1:25
CD31	C31.3+C31.7+C31.10	Novus Bio	NBP2-44342	68	24	Cy3	1:200
CD4	EPR6855	Abcam	ab181724	20	7	Cy3	1:100
CD44	IM-7	Novus Bio	NBP1-41266	44	14	Cy5	1:100
CD45	2B11+PD7/26	Novus Bio	NBP2-34287	56	25	Cy3	1:400
CD45RA	HI100	Biolegend	304102	72	13	Cy5	1:50
CD45RO	UCH-L1	Biolegend	304202	2	20	Cy3	1:100
CD5	UCHT2	Biolegend	300602	75	8	Cy3	1:50
CD56	MRQ-42	Cell Marque	156R	29	2	Cy5	1:50
CD57	HCD57	Biolegend	322325 (discontinued)	30	16	Cy3	1:200
CD68	KP-1	Biolegend	916104	70	21	Cy5	1:100
CD69	polyclonal	R&D Systems	AF2359	36	15	Cy3	1:200
CD7	MRQ-56	Cell Marque	107M	63	19	Cy3	1:100
CD8	C8/144B	Novus Bio	NBP-34588	8	15	Cy5	1:50
Collagen IV	polyclonal	Abcam		33	22	Cy5	1:200
Cytokeratin	C11	Biolegend	628601	67	20	Cy5	1:200
EGFR	D38B1	CST	26038	58	13	Cy3	1:25
FoxP3	236A/E7	Abcam	ab96048	61	3	Cy3	1:100
Granzyme B	EPR20129-217	Abcam	ab219803	81	7	Cy5	1:200
HLA-DR	EPR3692	Abcam	ab209968	65	17	Cy3	1:200
IDO-1	D5J4E	CST	91473	59	10	Cy5	1:25
LAG-3	D2G4O	CST	25848	42	8	Cy5	1:25
Mast cell tryptase	AA1	Abcam	ab2378	44	26	Cy3	1:200
MMP-9	L51/82	Biolegend	628602	80	24	Cy5	1:200
MUC-1	955	Novus Bio	NBP2-44658	15	6	Cy5	1:100
PD-1	D4W2J	CST	63815	23	9	Cy5	1:50
PD-L1	E1L3N	CST	85164	11	6	Cy3	1:50
Podoplanin	D2-40	Biolegend	916606	32	23	Cy5	1:200
T-bet	D6N8B	CST	27112	5	2	Cy3	1:100
TCRb	G11	Santa Cruz Biotech	sc-5277	3	12	Cy5	1:100
TCR-γ/δ	H-41	Santa Cruz Biotech	sc-100289	52	4	Cy3	1:100
Tim-3	polyclonal	R&D Systems	AF2365	21	3	Cy5	1:50
Vimentin	RV202	BD	550513	7	22	Cy3	1:200
VISTA	D1L2G	CST	82119	79	11	Cy5	1:50
α-SMA	polyclonal	Abcam	ab5694	69	26	Cy5	1:200
β-catenin	14	BD	610154	51	16	Cy5	1:50

Supplementary Table 3. Segmentation parameters

Methods	Tissue type	model_mpp*	maxima_threshold	interior_threshold	Nuclear channel	Membrane channel combination
MIBI	cHL 1	1.75 (0.6.0)	NA	NA	Histone H3	HLA-DR, HLA1, Na-K-ATPase, CD45RA, CD11c, CD3, CD20, CD68
MIBI	cHL 2	1.75 (0.6.0)	NA	NA	Histone H3	HLA-DR, HLA1, Na-K-ATPase, CD45RA, CD11c, CD3, CD20, CD68
CODEX	cHL	0.5 (0.12.2)	0.25	0.2	DAPI	CD4, CD7, CD15, CD30, CD11b, CD20, CD45RA, CD45RO, CD31, Podoplanin, HLA-DR

Note:
*deepcell-tf version and their weight file address
0.6.0: https://deepcell-data.s3-us-west-1.amazonaws.com/model-weights/Multiplex_Segmentation_20200908_2_head.h5
0.12.2: https://deepcell-data.s3-us-west-1.amazonaws.com/saved-models/MultiplexSegmentation-9.tar.gz

Supplementary Table 4. Confusion matrix of MAPS predictions across five folds on cHL1 MIBI dataset.

	**B**	**CD4 T**	**CD4 CTL**	**CD8 T**	**DC**	**Endothelial**	**M1**	**M2**	**NK**	**Neutrophil**	**Other**	**Treg**	**Tumor**
**B**	151670	1070	1518	678	1202	187	59	1406	480	23	761	732	2044
**CD4 T**	3768	320443	1073	12685	10517	621	104	7111	931	89	25713	2900	3945
**CD4 CTL**	294	1216	61072	1282	292	114	17	400	147	227	897	511	715
**CD8 T**	2201	9413	8343	198344	6761	341	663	6019	1200	980	3043	4241	4086
**DC**	462	1187	415	1650	95343	50	727	3850	2211	439	861	535	2042
**Endothelial**	6	6	40	10	1	19744	6	5	34	93	990	5	6
**M1**	7	0	3	13	15	7	13077	33	49	22	2	13	2
**M2**	1130	3143	2347	3899	1902	239	4530	192897	613	313	4820	1319	1142
**NK**	2	1	10	6	25	31	10	7	25270	15	1	13	4
**Neutrophil**	0	1	17	15	18	63	14	14	42	17794	33	18	216
**Other**	1041	2307	5079	1140	2414	695	26	1786	415	895	104558	418	4487
**Treg**	59	53	272	174	203	42	71	85	331	159	64	59736	224
**Tumor**	728	575	472	604	1050	51	17	231	139	2955	2275	1122	47698

Supplementary Table 5. Confusion matrix of CellSighter predictions across five folds on cHL1 MIBI dataset.

	**B**	**CD4 T**	**CD4 CTL**	**CD8 T**	**DC**	**Endothelial**	**M1**	**M2**	**NK**	**Neutrophil**	**Other**	**Treg**	**Tumor**
**B**	136217	3771	2316	3611	2060	645	108	3185	740	63	4918	1333	2863
**CD4 T**	9802	249282	5810	22158	24819	2726	299	17638	3180	341	37475	10336	6031
**CD4 CTL**	1078	3294	47750	4007	927	291	37	1593	479	896	3765	1496	1571
**CD8 T**	5328	18982	11394	166626	9798	879	381	11435	2085	1098	7918	5352	4354
**DC**	1325	4728	957	4069	82988	109	1204	5559	1958	348	2602	1673	2251
**Endothelial**	150	240	183	360	92	17637	40	216	64	281	1534	93	56
**M1**	59	58	38	331	703	16	8787	2633	49	45	387	105	32
**M2**	3632	8037	3666	10176	4933	560	6688	160223	937	510	14965	2207	1754
**NK**	322	493	282	714	2523	76	94	384	19193	83	558	536	137
**Neutrophil**	32	52	587	593	541	376	51	245	139	12793	911	430	1495
**Other**	3204	6872	4421	3404	3707	1691	633	7981	950	1854	84763	1192	4585
**Treg**	396	2101	720	5585	1302	143	64	1173	412	169	840	47779	788
**Tumor**	3314	1507	2254	2718	3860	239	79	1335	336	4078	5228	1839	31129

Supplementary Table 6. Confusion matrix of ASTIR predictions across five folds on cHL1 MIBI dataset.

	B	CD4 T	CD4 CTL	CD8 T	DC	Endothelial	M1	M2	NK	Neutrophil	Other	Treg	Tumor
**B**	67593	3140	29226	307	660	14	32045	915	57	3	26653	1217	0
**CD4 T**	279	49791	111612	704	1049	7	112562	418	79	1	111527	1869	2
**CD4 CTL**	135	17282	19316	104	17	4	19188	114	60	14	9959	991	0
**CD8 T**	433	53379	94219	14331	4137	37	49115	2651	404	48	26305	576	0
**DC**	292	18915	35250	528	22536	2	18002	584	36	232	13265	130	0
**Endothelial**	184	457	5896	27	24	7949	3758	81	11	816	1520	223	0
**M1**	63	363	1541	12	2689	2	5456	70	0	15	2923	109	0
**M2**	360	6566	59946	94	1837	26	55286	38812	28	73	54636	630	0
**NK**	331	4438	8712	174	2604	21	4402	216	6	74	3814	602	1
**Neutrophil**	10	1594	789	24	177	1	6027	149	1	6070	1828	1575	0
**Other**	211	8256	46427	29	299	5	34276	156	5	76	35124	397	0
**Treg**	73	9899	12868	1177	1030	4	852	870	36	51	317	34296	0
**Tumor**	1024	5252	16660	19	1683	3	20401	237	1	572	10055	2010	0

Supplementary Table 7. Confusion matrix of MAPS predictions across five folds on cHL2 MIBI dataset.

	B	CD4 T	CD8 T	DC	Endothelial	M1	M2	NK	Neutrophil	Other	Treg	Tumor
**B**	10020	132	70	127	10	21	63	18	39	211	210	471
**CD4 T**	407	29241	1175	1648	167	946	579	216	813	568	3296	717
**CD8 T**	27	126	10657	405	12	163	114	46	102	42	598	39
**DC**	84	349	283	13933	26	629	669	94	158	186	855	367
**Endothelial**	14	3	4	16	4250	7	7	6	15	31	14	1
**M1**	3	53	71	143	4	3658	102	13	17	16	36	21
**M2**	67	286	469	659	50	406	18972	91	770	1424	44	118
**NK**	10	7	19	58	19	10	49	3445	11	130	41	37
**Neutrophil**	22	108	121	81	55	33	176	34	15611	280	156	47
**Other**	47	63	37	163	113	25	303	258	802	12995	104	44
**Treg**	194	1550	475	989	151	203	73	120	165	376	32614	279
**Tumor**	210	98	39	80	1	23	68	61	39	65	92	6326

Supplementary Table 8. Confusion matrix of CellSighter predictions across five folds on cHL2 MIBI dataset.

	B	CD4 T	CD8 T	DC	Endothelial	M1	M2	NK	Neutrophil	Other	Treg	Tumor
**B**	8975	679	89	231	31	35	273	40	101	325	344	269
**CD4 T**	533	25749	1569	2103	201	586	1588	253	999	1729	4045	418
**CD8 T**	62	1280	8267	551	24	225	432	77	303	211	832	67
**DC**	193	2363	510	9786	60	557	1701	180	265	614	1254	149
**Endothelial**	50	162	27	87	3292	20	148	23	92	347	112	8
**M1**	14	661	231	572	24	1544	700	25	68	103	177	18
**M2**	162	1351	680	1174	98	425	15528	197	1331	2065	278	66
**NK**	45	288	91	219	42	8	250	2260	65	307	201	60
**Neutrophil**	49	708	181	325	73	47	731	38	13724	528	282	38
**Other**	138	792	51	493	251	45	2497	295	1160	8668	521	42
**Treg**	327	5916	1167	1821	202	195	339	193	199	860	25821	148
**Tumor**	713	631	63	309	9	13	133	113	113	148	131	4725

Supplementary Table 9. Confusion matrix of ASTIR predictions across five folds on cHL2 MIBI dataset.

	B	CD4 T	CD8 T	DC	Endothelial	M1	M2	NK	Neutrophil	Other	Treg	Tumor
**B**	4938	895	2	5114	0	16	10	130	62	27	28	170
**CD4 T**	185	13551	83	16197	18	20	185	154	92	64	2954	6270
**CD8 T**	38	2277	425	5327	5	48	74	179	38	78	671	3171
**DC**	151	4799	2	5308	4	251	192	1153	12	142	589	5030
**Endothelial**	39	506	5	2381	178	1	8	44	23	48	23	1112
**M1**	13	926	217	1126	1	85	145	286	0	14	62	1262
**M2**	240	1903	80	11338	3	533	1109	133	24	57	141	7795
**NK**	32	516	35	1772	0	6	18	25	11	235	133	1053
**Neutrophil**	15	1104	68	10879	8	79	149	31	1838	7	150	2396
**Other**	15	1436	1	8845	0	2	0	2	0	62	119	4472
**Treg**	141	8697	19	18529	25	82	38	837	29	210	5595	2987
**Tumor**	438	3311	5	2885	36	13	3	45	21	82	77	186

Supplementary Table 10. Confusion matrix of MAPS predictions across five folds on cHL CODEX dataset.

	B	CD4 T	CD8 T	DC	Endothelial	M1	M2	NK	Neutrophil	Other	Treg	Tumor	Epithelial	Lymphatic	Mast	Monocyte
**B**	13780	615	293	369	111	56	138	15	74	264	19	17	89	170	53	133
**CD4 T**	1158	28399	978	722	399	660	663	168	124	517	2121	289	405	298	169	410
**CD8 T**	171	300	16021	64	115	52	72	15	41	118	215	15	145	105	68	51
**DC**	162	188	58	7869	66	296	194	149	8	131	42	176	42	37	25	194
**Endothelial**	33	27	30	36	8168	9	41	29	41	185	7	2	19	8	15	55
**M1**	15	44	18	64	4	2784	95	3	3	9	11	5	7	3	4	32
**M2**	76	72	37	103	45	273	6197	13	12	235	34	7	33	24	20	105
**NK**	2	20	10	103	40	8	15	6394	232	6	55	21	14	4	10	405
**Neutrophil**	8	5	4	6	4	2	2	78	3232	6	4	27	1	3	2	58
**Other**	41	34	41	10	98	13	48	4	9	4682	9	23	30	14	33	19
**Treg**	4	248	44	18	7	4	28	25	4	8	2897	2	22	5	22	14
**Tumor**	3	46	15	106	0	8	9	22	76	23	4	7821	1	43	29	54
**Epithelial**	9	23	16	7	6	6	7	3	0	18	4	0	2130	3	4	15
**Lymphatic**	27	30	14	15	4	3	8	4	3	12	4	17	7	3596	6	18
**Mast**	6	3	9	5	6	4	6	2	1	9	5	6	4	1	3254	3
**Monocyte**	44	82	36	88	52	72	105	278	148	63	13	55	37	21	7	5812

Supplementary Table 11. Confusion matrix of CellSighter predictions across five folds on cHL CODEX dataset.

	B	CD4 T	CD8 T	DC	Endothelial	M1	M2	NK	Neutrophil	Other	Treg	Tumor	Epithelial	Lymphatic	Mast	Monocyte
**B**	12234	1343	585	518	161	47	245	30	81	362	24	28	87	182	88	181
**CD4 T**	1948	27637	1474	1024	467	547	688	241	131	714	832	323	347	280	293	534
**CD8 T**	529	1032	14460	152	171	52	185	53	82	215	57	38	136	140	160	106
**DC**	367	540	144	6579	156	317	292	230	22	172	28	326	54	72	44	294
**Endothelial**	129	270	202	129	6970	24	158	107	111	279	10	2	77	28	52	157
**M1**	48	311	50	291	24	1894	294	19	10	26	17	15	19	8	11	64
**M2**	204	316	136	249	151	240	5054	42	35	386	31	29	80	35	65	233
**NK**	31	167	69	291	90	15	42	5456	244	8	35	80	33	25	10	743
**Neutrophil**	82	54	53	26	95	14	34	250	2505	43	4	93	8	16	10	155
**Other**	223	296	174	131	311	19	250	11	28	3289	2	48	91	44	78	113
**Treg**	11	371	47	17	8	6	27	25	2	6	2797	5	8	2	16	4
**Tumor**	21	194	23	337	6	16	17	80	78	38	4	7216	4	57	58	111
**Epithelial**	69	240	119	56	54	10	53	20	6	66	10	4	1470	12	16	46
**Lymphatic**	99	99	81	46	17	4	33	20	16	33	5	63	19	3169	15	49
**Mast**	97	138	92	34	44	3	62	6	10	94	8	55	15	14	2628	24
**Monocyte**	152	339	96	396	129	71	259	864	200	161	12	138	58	63	25	3950

Supplementary Table 12. Confusion matrix of ASTIR predictions across five folds on cHL CODEX dataset.

	B	CD4 T	CD8 T	DC	Endothelial	M1	M2	NK	Neutrophil	Other	Treg	Tumor	Epithelial	Lymphatic	Mast	Monocyte
**B**	10608	244	23	209	17	197	220	431	12	3444	130	7	374	19	5	256
**CD4 T**	4685	17023	97	481	73	573	298	681	165	7878	3175	669	497	143	182	860
**CD8 T**	1815	1792	9199	114	48	100	192	69	35	3056	307	111	316	18	26	370
**DC**	224	554	22	1646	66	415	166	1225	380	2958	461	545	138	95	11	731
**Endothelial**	169	166	56	26	5189	91	36	24	205	2324	81	1	72	16	11	238
**M1**	27	388	17	1613	12	192	204	45	23	236	180	15	33	20	21	75
**M2**	23	122	6	265	85	608	4188	370	9	1416	64	3	82	2	6	37
**NK**	43	221	36	17	85	64	42	304	4994	327	63	71	195	136	13	728
**Neutrophil**	287	7	98	36	234	87	69	1	2004	229	20	35	32	50	3	250
**Other**	20	1	8	3	36	119	0	12	0	4641	61	1	163	3	6	34
**Treg**	41	1966	50	23	6	8	42	34	20	53	911	93	34	18	34	19
**Tumor**	16	6	2	18	0	34	5	23	25	1643	67	5565	6	196	347	307
**Epithelial**	6	179	14	3	42	1	37	0	0	8	1	0	1959	0	0	1
**Lymphatic**	138	43	207	3	43	11	9	17	0	362	54	0	47	2759	4	71
**Mast**	116	66	49	3	22	23	17	0	2	604	27	0	15	8	2310	62
**Monocyte**	153	136	14	15	41	656	111	195	1739	223	46	88	224	117	3	3152

Supplementary Table 13. Confusion matrix of MAPS predictions across five folds on DLBCL MIBI dataset.

	CD4 T	CD8 T	DC	M1	M2	NK	Other	Tumor	CD11b
**CD4 T**	16843	283	123	49	121	195	142	91	14
**CD8 T**	241	19106	92	76	147	141	22	67	12
**DC**	235	108	22317	592	35	210	84	112	60
**M1**	104	80	290	17517	139	124	6	110	29
**M2**	108	124	19	130	15729	109	36	33	45
**NK**	12	16	20	4	17	1182	6	7	1
**Other**	17	1	2	0	8	3	651	0	10
**Tumor**	1535	1574	1327	1609	872	2112	17	197415	188
**CD11b**	2	2	4	2	3	2	21	2	1541

Supplementary Table 14. Confusion matrix of CellSighter predictions across five folds on DLBCL MIBI dataset.

	CD4 T	CD8 T	DC	M1	M2	NK	Other	Tumor	CD11b
**CD4 T**	14660	1270	339	156	674	145	273	311	33
**CD8 T**	698	17952	130	181	549	58	34	270	32
**DC**	559	473	20070	1332	128	234	107	708	142
**M1**	234	230	667	15919	624	80	19	525	101
**M2**	197	237	56	157	15368	49	58	111	100
**NK**	63	19	66	14	23	984	7	76	13
**Other**	145	20	44	4	58	6	345	2	68
**Tumor**	3056	3310	2731	3154	2241	2306	30	189212	609
**CD11b**	19	27	26	27	50	9	57	21	1343

Supplementary Table 15. Confusion matrix of ASTIR predictions across five folds on DLBCL MIBI dataset.

	CD4 T	CD8 T	DC	M1	M2	NK	Other	Tumor	CD11b
**CD4 T**	5380	436	4978	0	3172	227	3016	0	652
**CD8 T**	817	6528	5046	42	1886	249	4309	0	1027
**DC**	864	209	1205	529	1679	206	15042	39	3980
**M1**	245	18	4178	3356	444	124	8813	11	1210
**M2**	563	240	6589	308	3573	98	4583	1	378
**NK**	20	15	432	1	201	324	185	6	81
**Other**	195	5	80	0	77	9	228	0	98
**Tumor**	2144	1119	76952	460	30483	2858	69987	6905	15741
**CD11b**	40	9	533	12	201	79	526	1	178
