# Supplementary material for: MAPS: pathologist-level cell type annotation from tissue images through machine learning
Source: Nat Commun. 2024 Jan 2;15:28. doi: 10.1038/s41467-023-44188-w (PMC10761896; doi:10.1038/s41467-023-44188-w)
Supplement: Supplementary file 3 — Reporting Summary [file 41467_2023_44188_MOESM3_ESM.pdf]

nature portfolio

Corresponding author(s): Sizun Jiang, Faisal, Mahmood

Last updated by author(s): Nov 10, 2023

Reporting Summary

Nature Portfolio wishes to improve the reproducibility of the work that we publish. This form provides structure for consistency and transparency in reporting. For further information on Nature Portfolio policies, see our Editorial Policies and the Editorial Policy Checklist.

Statistics

For all statistical analyses, confirm that the following items are present in the figure legend, table legend, main text, or Methods section.

n/a	Confirmed	
[ ]	[x]	The exact sample size (n) for each experimental group/condition, given as a discrete number and unit of measurement
[x]	[ ]	A statement on whether measurements were taken from distinct samples or whether the same sample was measured repeatedly
[x]	[ ]	The statistical test(s) used AND whether they are one- or two-sided<br>*Only common tests should be described solely by name; describe more complex techniques in the Methods section.*
[x]	[ ]	A description of all covariates tested
[x]	[ ]	A description of any assumptions or corrections, such as tests of normality and adjustment for multiple comparisons
[ ]	[x]	A full description of the statistical parameters including central tendency (e.g. means) or other basic estimates (e.g. regression coefficient) AND variation (e.g. standard deviation) or associated estimates of uncertainty (e.g. confidence intervals)
[x]	[ ]	For null hypothesis testing, the test statistic (e.g. F, t, r) with confidence intervals, effect sizes, degrees of freedom and P value noted<br>*Give P values as exact values whenever suitable.*
[x]	[ ]	For Bayesian analysis, information on the choice of priors and Markov chain Monte Carlo settings
[x]	[ ]	For hierarchical and complex designs, identification of the appropriate level for tests and full reporting of outcomes
[x]	[ ]	Estimates of effect sizes (e.g. Cohen's d, Pearson's r), indicating how they were calculated

Our web collection on statistics for biologists contains articles on many of the points above.

Software and code

Policy information about availability of computer code

Data collection	MIBI acquisition was performed on a commercially available MIBIscope System from Ionpath with MIBIcontrol v.1.7.0-0f60ffbc, image processing was performed using the toffy package (https://github.com/angelolab/toffy).<br>CODEX data were acquired on the CODEX microfluidics instrument with CODEX driver software; Multiplexed images were stitched and background corrected using the Singer software (v1.0.7) from Akoya.
Data analysis	Code for MAPS analysis will be public at https://github.com/mahmoodlab/MAPS, also on Zenodo: 10.5281/zenodo.10067742<br>Python pre-requisites for the MAPS package:<br>Python (3.9.0), PyTorch (1.13.1), Numpy (1.12.5), Pandas (1.5.5), Scikit-Learn (1.2.1), Mlxtend (0.22.0), Setuptools (63.4.1)<br><br>The pre-processing of the MIBI and CODEX data applied the following packages:<br>toffy (0.1.2), deepcell (0.6.0, 0.12.2), FlowSOM (2.4.0), Leiden (0.8.10)

For manuscripts utilizing custom algorithms or software that are central to the research but not yet described in published literature, software must be made available to editors and reviewers. We strongly encourage code deposition in a community repository (e.g. GitHub). See the Nature Portfolio guidelines for submitting code & software for further information.

Data

Policy information about availability of data

All manuscripts must include a data availability statement. This statement should provide the following information, where applicable:

- Accession codes, unique identifiers, or web links for publicly available datasets
- A description of any restrictions on data availability
- For clinical datasets or third party data, please ensure that the statement adheres to our policy

All the multiplexed imaging data described in this work, including channel images, segmentation masks and extracted CSVs, are available at Zenedo, with DOI: 10.5281/zenodo.10067009.

We also used publicly available dataset:
Schürch et al. Human CRC CODEX: DOI: 10.17632/mpjzbtfgfr.1
Wright et al. Human DLBLC MIBI: DOI: https://doi.org/10.1182/bloodadvances.2023009813

Research involving human participants, their data, or biological material

Policy information about studies with human participants or human data. See also policy information about sex, gender (identity/presentation), and sexual orientation and race, ethnicity and racism.

Reporting on sex and gender	Sex and gender were not considered in the study design due to the proof-of-concept nature of this methodological study.
Reporting on race, ethnicity, or other socially relevant groupings	Race, ethnicity, or other socially relevant groupings information were not considered in the study design due to the proof-of-concept nature of this methodological study.
Population characteristics	All patient samples used in this study were previously acquired for clinical related projects, and the main purpose to for this paper is to demonstrate the ability of the MAPS methodology. Thus, no population characteristics were taken into consideration for this project.
Recruitment	All patient samples used in this study were previously acquired for clinical related projects, and the main purpose to for this paper is to demonstrate the ability of the MAPS methodology. Thus, recruitment-related information is not part of our consideration for this methodology oriented paper.
Ethics oversight	All tissues were retrieved from the archives of Brigham and Women's Hospital (Boston, MA) with institutional review board approval (IRB# 2010P002736) and patient wavier of consent.

Note that full information on the approval of the study protocol must also be provided in the manuscript.

Field-specific reporting

Please select the one below that is the best fit for your research. If you are not sure, read the appropriate sections before making your selection.

☒ Life sciences ☐ Behavioural & social sciences ☐ Ecological, evolutionary & environmental sciences

For a reference copy of the document with all sections, see nature.com/documents/nr-reporting-summary-flat.pdf

Life sciences study design

All studies must disclose on these points even when the disclosure is negative.

Sample size	No sample size calculation was performed because this manuscript focuses on demonstration of MAPS methodology development. But to computationally benchmark MARIO and other methods, we selected sufficient dataset and cell number that is manageable in terms of time and computational power for different methods. Specifically, for each dataset we have 150k to 1.5 million cells with each subpopulation has high enough numbers of cell to be sufficiently represented during testing.
Data exclusions	No data were excluded from the analyses.
Replication	The replication of the MAPS methodology was verified in multiple datasets (two cHL-MIBI, one cHL-CODEX and one CRC-CODEX), imaging modalities (MIBI and CODEX). All the multiplexed cHL tissue sections (including the lymph node control) were stained with the same antibody cocktail, then imaged on the same MIBIscope machine.
Randomization	Randomization was not necessary for this study. Randomization is not relevant for this study because the experiments were aiming to validate technical performance.
Blinding	Blinding was not necessary for this study. Blinding is not relevant for this study because the experiments were aiming to validate technical performance.

Reporting for specific materials, systems and methods

We require information from authors about some types of materials, experimental systems and methods used in many studies. Here, indicate whether each material, system or method listed is relevant to your study. If you are not sure if a list item applies to your research, read the appropriate section before selecting a response.

Materials & experimental systems

n/a	Involved in the study
☐	☒ Antibodies
☒	☐ Eukaryotic cell lines
☒	☐ Palaeontology and archaeology
☒	☐ Animals and other organisms
☒	☐ Clinical data
☒	☐ Dual use research of concern
☒	☐ Plants

Methods

n/a	Involved in the study
☒	☐ ChIP-seq
☒	☐ Flow cytometry
☒	☐ MRI-based neuroimaging

Antibodies

Antibodies used

All clones, titers, and conjugation information can be found in Supplementary Information, Supplementary Table 1, MIBI panel for cHL 1 and cHL 2 (MIBI), and Supplementary Table 2, CODEX panel for cHL.

For the MIBI panel:
Antibody target Clone Oligo Cycle Fluoresecence Titer (dilution) Note
T-bet D6N8B 5 2 Cy3 1:100
FoxP3 236A/E7 61 3 Cy3 1:100
TCR-γ/δ H-41 52 4 Cy3 1:100
CD30 BerH2 57 5 Cy3 1:25
PD-L1 E1L3N 11 6 Cy3 1:50
CD4 EPR6855 20 7 Cy3 1:100
CD5 UCHT2 75 8 Cy3 1:50
CD25 4C9 24 9 Cy3 1:100
CD20 rIGEL/773 48 10 Cy3 1:200
CD11c EP1347Y 49 11 Cy3 1:50
CD16 D1N9L 26 12 Cy3 1:100
EGFR D38B1 58 13 Cy3 1:25
CD206 polyclonal 55 14 Cy3 1:100
CD69 polyclonal 36 15 Cy3 1:200
CD57 HCD57 30 16 Cy3 1:200
HLA-DR EPR3692 65 17 Cy3 1:200
BCL-2 124 41 18 Cy3 1:50
CD7 MRQ-56 63 19 Cy3 1:100
CD45RO UCH-L1 2 20 Cy3 1:100
CD163 EDHu-1 45 21 Cy3 1:200
Vimentin RV202 7 22 Cy3 1:200
CD15 MMA 14 23 Cy3 1:200
CD31 C31.3+C31.7+C31.10 68 24 Cy3 1:200
CD45 B11+PD7/26 56 25 Cy3 1:400
Mast cell tryptase AA1 44 26 Cy3 1:200
CD56 MRQ-42 29 2 Cy5 1:50
Tim-3 polyclonal 21 3 Cy5 1:50
CCR6 polyclonal 53 4 Cy5 1:25
CD2 RPA-2.10 25 5 Cy5 1:25
MUC-1 955 15 6 Cy5 1:100
Granzyme B EPR20129-217 81 7 Cy5 1:200
LAG-3 D2G4O 42 8 Cy5 1:25
PD-1 D4W2J 23 9 Cy5 1:50
IDO-1 D5J4E 59 10 Cy5 1:25
VISTA D1L2G 79 11 Cy5 1:50
TCRb G11 3 12 Cy5 1:100
CD45RA HI100 72 13 Cy5 1:50
CD44 IM-7 44 14 Cy5 1:100
CD8 C8/144B 8 15 Cy5 1:50
β-catenin 14 51 16 Cy5 1:50
CD11b EPR1344 28 19 Cy5 1:50

Cytokeratin C11 67 20 Cy5 1:200
CD68 KP-1 70 21 Cy5 1:100
Collagen IV polyclonal 33 22 Cy5 1:200
Podoplanin D2-40 32 23 Cy5 1:200
MMP-9 L51/82 80 24 Cy5 1:200
CD162 HECA-452 46 25 Cy5 1:200
α-SMA polyclonal 69 26 Cy5 1:200

For the CODEX panel:
Antibody target Clone Vendor Catalog number Oligo Cycle Fluor Titer (dilution)
BCL-2 124 Novus Bio NBP2-34443 41 18 Cy3 1:50
CCR6 polyclonal Novus Bio NBP1-88565 53 4 Cy5 1:25
CD11b EPR1344 Abcam ab209970 28 19 Cy5 1:50
CD11c EP1347Y Abcam ab216655 49 11 Cy3 1:50
CD15 MMA BD 559045 14 23 Cy3 1:200
CD16 D1N9L CST 72204 26 12 Cy3 1:100
CD162 HECA-452 Novus Bio NB100-78039 46 25 Cy5 1:200
CD163 EDHu-1 Novus Bio NB110-40686 45 21 Cy3 1:200
CD2 RPA-2.10 Biolegend 300202 25 5 Cy5 1:25
CD20 rIGEL/773 Novus Bio NBP2-53190 48 10 Cy3 1:200
CD206 polyclonal R&D Systems AF2535 55 14 Cy3 1:100
CD25 4C9 Cell Marque 125M 24 9 Cy3 1:100
CD30 BerH2 Cell Marque 130M 57 5 Cy3 1:25
CD31 C31.3+C31.7+C31.10 Novus Bio NBP2-44342 68 24 Cy3 1:200
CD4 EPR6855 Abcam ab181724 20 7 Cy3 1:100
CD44 IM-7 Novus Bio NBP1-41266 44 14 Cy5 1:100
CD45 2B11+PD7/26 Novus Bio NBP2-34287 56 25 Cy3 1:400
CD45RA HI100 Biolegend 304102 72 13 Cy5 1:50
CD45RO UCH-L1 Biolegend 304202 2 20 Cy3 1:100
CD5 UCHT2 Biolegend 300602 75 8 Cy3 1:50
CD56 MRQ-42 Cell Marque 156R 29 2 Cy5 1:50
CD57 HCD57 Biolegend 322325 (discontinued) 30 16 Cy3 1:200
CD68 KP-1 Biolegend 916104 70 21 Cy5 1:100
CD69 polyclonal R&D Systems AF2359 36 15 Cy3 1:200
CD7 MRQ-56 Cell Marque 107M 63 19 Cy3 1:100
CD8 C8/144B Novus Bio NBP-34588 8 15 Cy5 1:50
Collagen IV polyclonal Abcam 33 22 Cy5 1:200
Cytokeratin C11 Biolegend 628601 67 20 Cy5 1:200
EGFR D38B1 CST 26038 58 13 Cy3 1:25
FoxP3 236A/E7 Abcam ab96048 61 3 Cy3 1:100
Granzyme B EPR20129-217 Abcam ab219803 81 7 Cy5 1:200
HLA-DR EPR3692 Abcam ab209968 65 17 Cy3 1:200
IDO-1 D5J4E CST 91473 59 10 Cy5 1:25
LAG-3 D2G4O CST 25848 42 8 Cy5 1:25
Mast cell tryptase AA1 Abcam ab2378 44 26 Cy3 1:200
MMP-9 L51/82 Biolegend 628602 80 24 Cy5 1:200
MUC-1 955 Novus Bio NBP2-44658 15 6 Cy5 1:100
PD-1 D4W2J CST 63815 23 9 Cy5 1:50
PD-L1 E1L3N CST 85164 11 6 Cy3 1:50
Podoplanin D2-40 Biolegend 916606 32 23 Cy5 1:200
T-bet D6N8B CST 27112 5 2 Cy3 1:100
TCRb G11 Santa Cruz Biotech sc-5277 3 12 Cy5 1:100
TCR-γ/δ H-41 Santa Cruz Biotech sc-100289 52 4 Cy3 1:100
Tim-3 polyclonal R&D Systems AF2365 21 3 Cy5 1:50
Vimentin RV202 BD 550513 7 22 Cy3 1:200
VISTA D1L2G CST 82119 79 11 Cy5 1:50
α-SMA polyclonal Abcam ab5694 69 26 Cy5 1:200
β-catenin 14 BD 610154 51 16 Cy5 1:50

Validation

All multiplexed staining images were compared to online databases, including the Human Protein Atlas. They are then reviewed by at least 3 separate individuals, including a board certified pathologist or subject matter expert for consensus-based quality control.
All antibody clones used in this study have also been cross validated with the staining pattern in previous published multiplexed imaging studies by our laboratories (Jiang, S.Z., et al., Immunity, 2022; Phillips, D., et al., Frontiers in Immunology, 2021).
